# Clinicopathologic, treatment and prognosis study of 46 Xp11.2 translocation/TFE3 gene fusion renal cell carcinomas

**DOI:** 10.1186/s12894-022-01060-1

**Published:** 2022-07-18

**Authors:** Jiale Zhou, Ling Zhao, Zhaolin Yang, Yonghui Chen, Xiaorong Wu, Wei Xue

**Affiliations:** 1 Department of Urology, Renji Hospital, School of Medicine, Shanghai Jiaotong University, Shanghai, 200127 China; 2 Department of Pathology, Renji Hospital, School of Medicine, Shanghai Jiaotong University, Shanghai, 200127 China

**Keywords:** Xp11.2, Translocational renal cell carcinoma, *TFE3* gene, RCC

## Abstract

**Purpose:**

To report the clinicopathological features and mid- to long-term oncologic results of Xp11.2 translocation/transcription factor E3 (*TFE3*) gene fusion renal cell carcinomas (Xp11.2 translocation RCCs) in a single large-volume centrecentre.

**Methods:**

Clinical and follow-up data of 46 patients who were diagnosed with Xp11.2 translocation RCC and underwentunderwent surgical intervention were retrospectively reviewed.

**Result:**

Forty-six Xp11.2 translocation RCC patients were identified from 4218 renal tumour patients who were underwentunderwent surgery in our centrecentre from Jan. 2014 to Apr. 2020. The incidence of Xp11.2 translocation RCCs in our centre was 1.09%. During a median follow-up period of 30.5 months, 4 patients died of the disease. The total median overall survival and cancer specific survival were 30.0 months and 24.0 months, respectively. The 1-year, 3-year and 5-year OS rates were 97.4%, 88.8%, and 88.8%, respectively. In multivariable analysis, displaying symptoms when diagnosed (*p* = 0.019), lymph node metastasis (*p* = 0.002) and distal metastasis (*p* = 0.020) were identified as risk factors for poor prognosis.

**Conclusion:**

Xp11.2 translocation RCC is a type of renal cell carcinoma with a relatively low incidence and various prognoses. Early-stage Xp11.2 translocation RCCs have a similar prognosis to most typical RCCs, but late-stage Xp11.2 translocation RCCs can lead to poor oncological outcomes.

## Introduction

Xp11.2 translocation renal cell carcinoma (Xp11.2 translocation RCC), first classified by WHO in 2004, is characterized by different tanslocations involving chromosome Xp11.2 and consequently results in gene fusions of the transcription factor E3 gene (*TFE3*) [1]. The tumour is frequently diagnosed in children and teenagers, accounting for approximately 20 ~ 40% of all renal malignancies in children, but rarely occurs in adults with merely 1 ~ 1.6% [2]. Although the total number of adult Xp11.2 translocation RCC patients still remains high due to the large population, the prognosis of the disease remains controversial. Previous studies reported a relatively better prognosis in children than in adults [3]. However, a recent meta-analysis of 15 studies reviewing 147 Xp11.2 translocation RCC patients demonstrated no significant difference observed in prognosis between children and adults, or between males and females [4]. On this basis, the main purpose of this retrospective study was to provide mid- to long-term survival data from a large cohort from a single centre and to analyse the potential risk factors for a poor prognosis of Xp11.2 translocation RCCs.

## Methods and materials

### Patient selection

A retrospective was performed for all the Xp11.2 translocation RCC patients treated at Renji Hospital, School of Medicine, Shanghai Jiaotong University from January 2014 to April 2020. The diagnosis and grading criteria of Xp11.2 translocation RCC in the current study were based on the 2016 WHO classification [5]. Clinical information including age, sex, symptoms of onset, tumour diameter, tumour location, pathological information, and surgical information was reviewed. Follow-up data were collected for the original surgeons.

Tumour size and gross appearance were obtained from the original pathologic reports, while the TNM stage was assessed according to the 8th edition of AJCC TNM staging [6]. Paraffin sections of all selected cases were reviewed by two independent pathologists (ZW and QL) for re-evaluation of nuclear grade according to ISUP criteria, as well as the histological pattern [7].

Statistical analysis was conducted using SPSS® version 20.0 (IBM Corp., Armonk, NY, United States) and GraphPad Prism 6 (San Diego, CA, United States). Data are presented as the median ± SD. Overall survival and cancer-specific survival were calculated with Kaplan–Meier curves. A Cox proportional hazards model was used to identify the prognostic factors by univariable analysis. *p* values (two-sided) less than 0.05 were considered statistically significant.

## Results

Forty-six patients were identified from 4218 patients who underwentunderwent partial or radical nephrectomy in Renji Hospital.

### Clinical information

The clinical data are summarized in Table 1. In total 46 patients were included in the current cohort, including nineteen male patients and twenty-seven female patients. The mean age was 39 years (ranging from 15 to 72). The tumour was accompanied by clinical symptoms at the time of diagnosis in 9 patients, which including four patients who complained of lumbar pain, four patients with haematuria and one patient with severe pelvic pain due to metastatic lesions. None of these patients in the cohort had developed any other malignancies before. However, two patients had lymphatic metastasis, one had bone metastasis, one had lung metastasis and one had both lymphatic and hepatic metastasis before surgery.

**Table 1:** Clinicopathological features of 46 Xp11.2 RCC patients

Clinicopathological features	n (%)
Age (yr)	
< 65/ ≥ 65	40 /6
Median (range)	39 (15–72)
Gender	
Male/female	19/27
Tumor location	
Left/right	27/19
Tumor size (max diameter, cmmm)	
Mean ± SD	41.29 ± 19.08
Symptoms (%)	
Lumbar pain	4 (8.9)
Pelvic pain	1 (2.1)
Hematuria	4 (8.9)
T classification	
T1a/T1b	13/8
T2a/T2b	3/0
T3	3
T4	0
Lymph node metastasis	
N0/N1	44/2
Distal metastasis	
M0/M1	43/3
Operation	
Radical nephrectomy	13
Partial nephrectomy	30
Cytoreduction surgery	3
Follow-up	
Median OS (mo)	30.0
Dead/alive/lost	4/36/6
Median CSS (mo)	24.0

*OS* Overall survival; *CSS* Cancer specific survival

Sixteen patients underwentunderwent radical nephrectomy, including three cytoreductive surgeries, while thirty patients received partial nephrectomy either by laparoscopic or open approaches. Retroperitoneal lymph node dissection was performed for patients with suspected lymphatic metastasis or a rather large tumour size (≥ cT2).

### Pathological characteristics

All tumours examined were unilateral and unifocal. The maximum diameter of the tumours ranged from 12 to 109 mm, and the median maximum diameter was 40 mm (IQR 18.5 ~ 60.5).

**Fig. 1:**
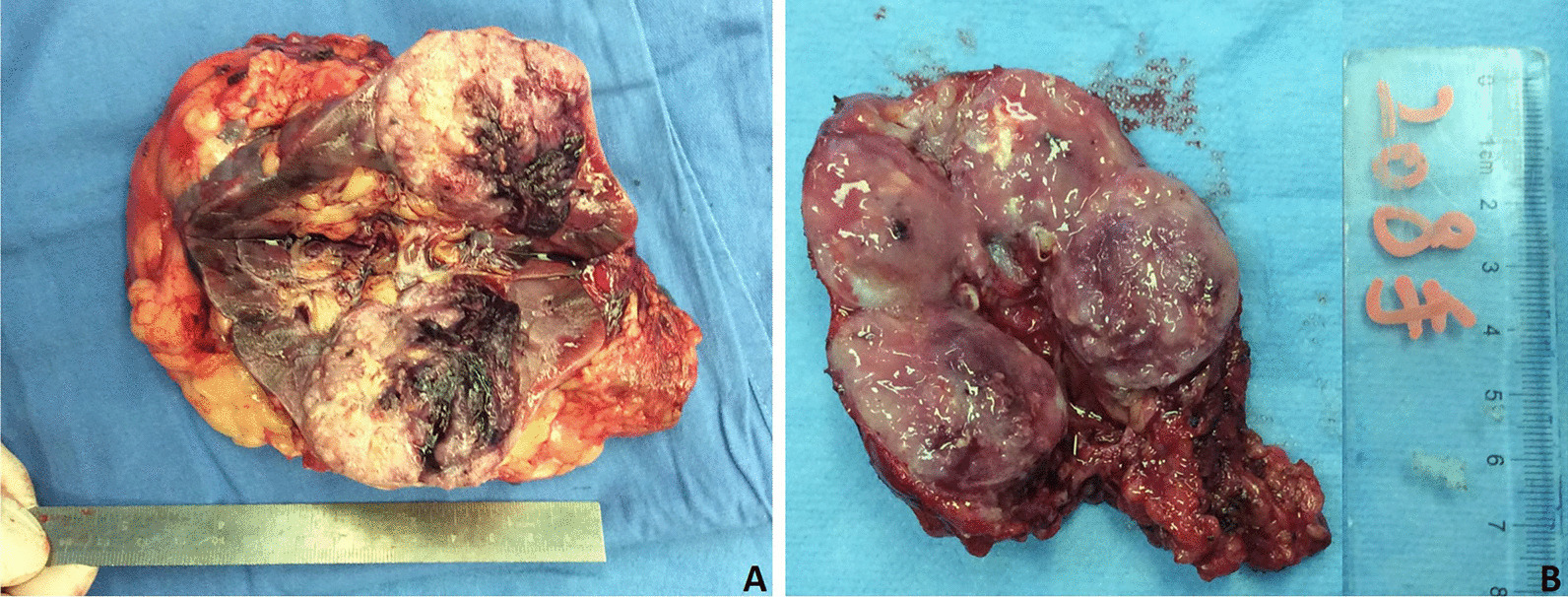
The gross appearance of typical Xp11.2 translocation RCC. **A** The tumour arose in the renal cortex with a yellowish cut surface with haemorrhage. **B** The tumour was well circumscribed and solid. The cut surface showed a light brown colour with necrosis

Most tumours arose in the renal cortex, with soft or firm texture. Although there was no distinctive gross appearance, in most Xp11.2 translocation RCC cases, the cut surface colouration was usually yellowish, which was different from the typical golden yellow colour in clear cell carcinoma (Fig. 1). Beyond that, haemorrhages and necrosis were occasionally noted.

**Fig. 2:**
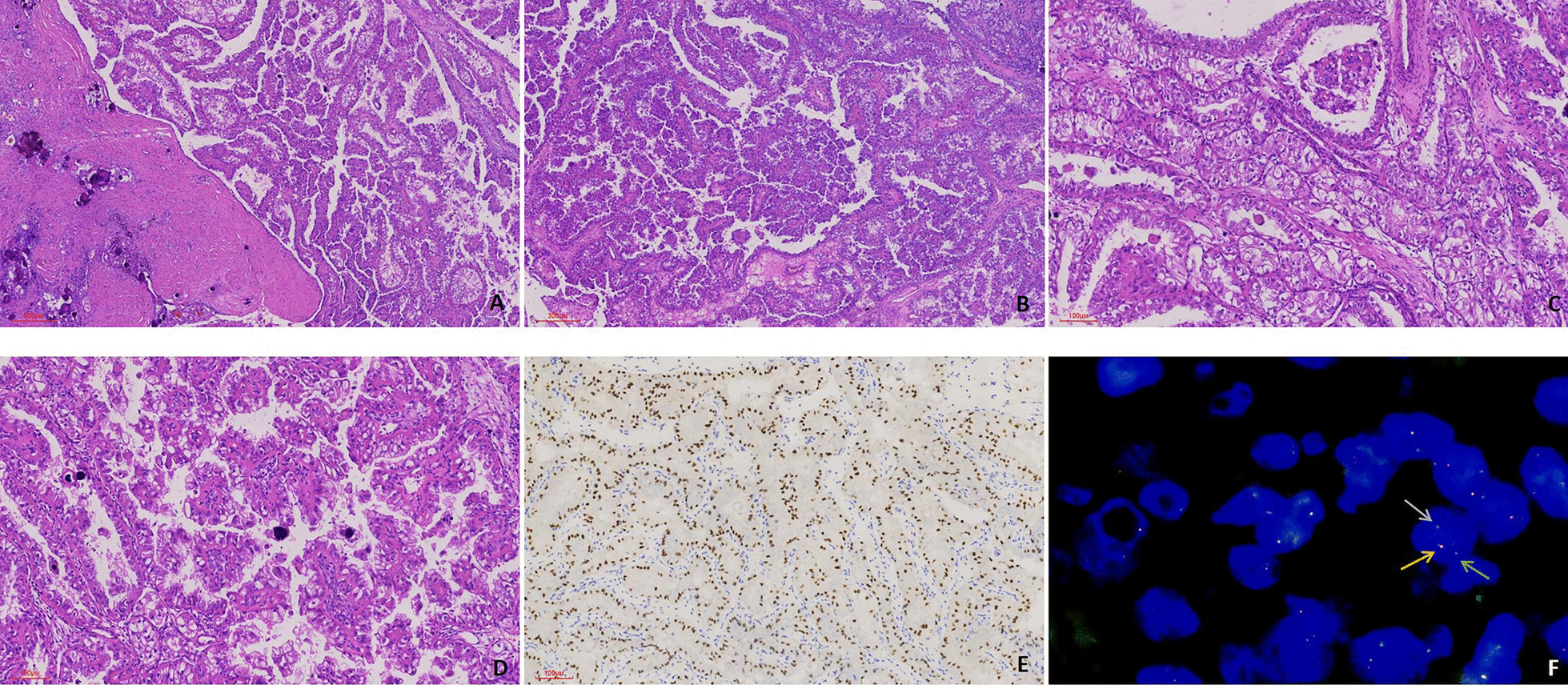
Representative pathologic image of Xp11.2 translocation RCC. **A** Tumour composed of clear and eosinophilic cells with psammoma bodies. **B** A neoplasm with a tubular and papillary structures. **C** Tumour cells with clear cytoplasm formed tubules with abundant psammoma bodies resembling a clear cell renal cell carcinoma. **D** Diffuse nuclear staining was detected. **E** The nucleus showed diffuse, strong staining for TFE3. **F** The demonstration of TFE3 gene rearrangement by FISH. One fused signal (yellow arrow) and two split signals (green and red arrow). (**A**–**C** Magnification: × 40; **D**–**E** Magnification: × 100; **F**: Magnification: × 1000)

In most cases, the histologic appearance of Xp11.2 translocation RCC is a neoplasm with mixed tubular and papillary structure, with cells of different morphology including clear or eosinophilic cytoplasm. Psammoma bodies, lymphocytes infiltration, and necrosis were found in most Xp11.2 translocation RCCs (Fig. 2A–E).

In the current cohort, all cases showed positive *TFE3* staining. Diffuse nuclear staining was detected under microscope. The nuclei showed diffuse, strong staining for *TFE3*.

In FISH analysis, *TFE3* gene rearrangement was detected by using a dual-colour break-apart FISH probe (Anbiping Inc., Grangzhou, China) according to the manufacturer's recommendations (Fig. 2F).

### Follow-up results

Patients were followed up for 4 to 74 months, with a median follow-up duration of 30.5 months. By the ultimate follow-up deadline, 6 patients had tumour recurrence or metastasis, among whom 4 patients died of the disease. The total median overall survival and cancer specific survival were 30.0 months and 24.0 months, respectively. The 1-year, 3-year and 5-year OS rates were 97.4%, 88.8%, and 88.8%, respectively.

In patient #4, who underwentunderwent laparoscopic partial nephrectomy for a 68 mm tumour with a negative surgical margin in pathologic diagnosis, local recurrence was found 4 months after surgery, and underwent another radical nephrectomy. The patient remained alive untill the end of follow-up. In patient #25, who underwent laparoscopic partial nephrectomy for a 32 mm tumour, lung metastasis was found 6 months after surgery. The patient received Axitinib for targeted therapy thereafter but still died 24 months after the initial surgery. Patient #29 was a pT1aN0M1 patients with bone metastasis who underwent both laparoscopic partial nephrectomy and resection of bone metastatic lesions. The patient received Pazopanib one month after surgery and combined with Nivolumab since 2019. The patient remained alive untill the end of follow-up. In patient #37, a T1aN0M0 patient underwent laparoscopic partial nephrectomy, multifocal metastases including lung, bone and lymph nodes were found 22 months after surgery. The patient received targeted therapy and died 34 months postoperatively. In patient #44, a pT1bN1M1 patient with liver and lymph node metastasis, multifocal metastasis was found in lung and liver shortly after cytoreductive nephrectomy, and the patient died 8 months after surgery. In patient #46, a pT2aN0M1 patient with lung metastasis treated with cytoreductive nephrectomy, the lung metastatic focus was enlarged despite of target therapy, and the patient died 10 months after surgery.

By evaluating potential survival factors, such as age, sex, tumour location, tumour size, T classification, lymph node metastasis, distal metastasis utilizing univariable Cox regression analysis and Kaplan–Meier analysis, we found that patients with symptoms when diagnosed (*p* = 0.019), lymph node metastasis (*p* = 0.002) and distal metastasis (*p* = 0.020) were associated with a poor oncologic outcome.

## Discussion

In the 2004 WHO classification of renal tumours, renal carcinoma associated with Xp11.2 translocational/*TFE3* gene fusions (Xp11.2 translocation RCCs) was first accepted as a distinct subtype of RCC [1]. Xp11.2 translocation RCC is characterized by the translocation on chromosome Xp11.2 and a gene fusion between *TFE3* and several different genes, including *ASPL* (17q25), *CLTC* (17q23), *NonO* (Xp12), *PSF* (1q34), and *PRCC* (1q21) [8]. Previous reports demonstrated that the overall incidence of Xp11.2 translocation RCC is low and mainly occurs in children and young people [2]. However, due to the relatively low incidence, the population characteristics, clinical characteristics and oncological outcomes remain controversial [4, 9]. On this bias, we present the clinical findings and follow-up results of 46 Xp11.2 translocation RCC patients in our centre.

The incidence of Xp11.2 translocation RCCs in our centre was 1.09%, which is coincident with the 1% to 1.6% occurrence reported before [2, 8, 9]. During a median follow-up period of 30.5 months, 4 patients died of the disease. The total median overall survival and cancer specific survival were 30.0 months and 24.0 months, respectively. The 1-year, 3-year and 5-year OS rates were 97.4%, 88.8%, and 88.8%.

Traditionally, Xp11.2 translocation RCC is associated with advanced tumour stage and poor oncological outcome due to aggressive biological behavior [10]. In previous studies, more than half of Xp11.2 translocation RCC patients were found to have metastatic lesions and consequently died of the disease [11, 12]. However, in our cohort, only 4 patients displayed lymph node or distal metastasis at the time of diagnosis.

There seem to be no specific macroscopic appearance characteristics of Xp11.2 translocation RCCs. In the current study, most tumour presented a gross appearance of a sulfur yellow to grey cut surface along with haemorrhage and necrosis, which is commonly found in clear cell carcinomas. The microscopic appearance includes papillary, tubular, nested and mixed patterns. In our study, we also found the presence of clear cells in several cases, which was also reported previously. One recognizable microscopic finding of Xp11.2 translocation RCCs is the presence of psammoma bodies. TFE 3 immunostaining is the most commonly used method for diagnosing Xp11.2 translocation RCCs. According to the literature, the specificity and sensitivity of IHC was 99.6% and 97.5%, respectively. [13] Previously, many Xp11.2 translocation RCCs were misdiagnosed as type II pRCCs or ccRCCs due to limited understanding and heterogeneous pathologic appearance. Now, with the introduction of the FISH assay in 2011, the *TFE3* break-apart FISH assay has become indispensable diagnostic method for Xp11.2 translocation RCCs [14, 15]. The diagnosis of 46 Xp11.2 translocation RCCs in the current study was confirmed by FISH analysis.

In the current study, we report 46 Xp11.2 translocation RCC patients with 1-year, 3-year and 5-year OS rates of 97.4%, 88.8%, and 88.8%, respectively. The result indicates a considerable long-term survival, which approximately equals the 5-year overall survival of RCCs. (SEER Cancer Statistics Review, 1975–2014, based on November 2016 SEER data submission, posted to the SEER website, April 2017: National Cancer Institute.) The same follow-up outcome was also reported by Klatte et al., who identified 2 Xp11.2 translocation RCCs from 848 RCC cases through FISH and PCR [2]. However, the current existing large-scale studies report poorer overall survival and cancer-specific survival of Xp11.2 translocation RCCs than non-Xp11.2 translocation RCCs [9–12]. Regarding the contrast between two different outcomes, by reviewing the cohort, one possible reason we found may be the high proportion of cT1 Xp11.2 tumours in the current cohort. In previous studies, the reported approximate proportion of cT1 ~ 2 stage was 36.4% ~ 64.4% [10, 11, 16], however, this rate in current study was 91.1%. This may be probably due to the development of imaging devices that enables the early detection of RCCs in their early stages. This phenomenon may also indicate that as long as treated at its early stage (cT1 ~ 2), Xp11.2 translocation RCCs can present as good a prognosis as other types of RCCs.

Since the translocation happens on the X chromosome, there exists the hypothesis that the Xp11.2 translocation RCCs may have a gender-related predominance in the incidence in females [17]. In our study, the same result was reported. Beyond that, in multifactor analysis, there was no significant difference between females and males in the incidence of lymph node metastasis and distal metastasis and overall survival, which is similar to the report of Cheng et al.^17^. Furthermore, by evaluating the potential risk factors for poor prognosis, we identified independent risk factors including displaying symptoms when diagnosed (*p* = 0.019), lymph node metastasis (*p* = 0.002) and distal metastasis (*p* = 0.020). The result is also coincident with those in other types of RCCs.

However, there are still limitations in the current study. Even though the current cohort is one of the largest Xp11.2 translocation RCC cohorts, the number is still too insufficient to draw decisive conclusions. The retrospective and single-centre design of the study also led to many biases. Therefore, large-scale multicentre studies with more detailed clinical and laboratory information are required in the future.

In summary, we reported mid-to-long-term oncological outcomes of a 46-patient Xp11.2 translocation RCC cohort from a single centre and identified some prognostic clinical factors. By reviewing previous studies of Xp11.2 translocation RCCs and other non-Xp11.2 translocation RCC types, we came to the primary conclusion that Xp11.2 translocation RCCs found and treated in their early stages may have as good a prognosis as many other types of RCCs. On the other hand, Xp11.2 translocation RCCs with clinical symptoms, regional lymph node metastasis or distal metastasis may have relatively poor clinical outcomes and are not sensitive to VEGF-targeted therapy, but ICI may have a potential therapeutic effect.

## Data Availability

The datasets from this study can be found in the cited guidelines. Analyses can be obtained from corresponding author upon request.
